# Supplementary material for: Are carbon emissions trading and green financial instruments synergistic? -Comprehensive quantitative research based on content analysis
Source: PLoS One. 2024 Mar 7;19(3):e0298601. doi: 10.1371/journal.pone.0298601 (PMC10919601; doi:10.1371/journal.pone.0298601)
Supplement: S1 Data — (ZIP) [file pone.0298601.s001.zip › Data/地级市Z_F工作报告.zip!/░▓╗╒/═¡┴Ω╩╨/└┤╫╘╣1⁄2╓┌║┼í╛┬φ┐╦╩2╛▌═°í┐_2015.docx]

各位代表：

    现在，我代表市人民政府，向大会报告工作，请予审议，并请市政协委员和其他列席人员提出意见和建议。

    一、2014年工作回顾

    刚刚过去的一年，宏观环境复杂严峻，经济下行压力持续加大。全市人民在中共铜陵市委的坚强领导下，认真贯彻落实中央和省委、省政府决策部署，坚持稳中求进，奋力克难攻坚，统筹做好稳增长、调结构、促改革、惠民生、防风险各项工作，经济发展稳中有进、稳中向好，各项事业全面进步，社会大局和谐稳定。预计，全年实现地区生产总值715亿元，增长10%，增幅居全省前列；财政收入132亿元，增长1.5%，其中地方收入增长3%；全社会固定资产投资767亿元，增长18%；社会消费品零售总额175亿元，增长12.5%；全市常住居民人均可支配收入25057元，增长9.9%，总量居全省第一；新增就业2.61万人；居民消费价格上涨1.1%；人口自然增长率2.78％。；节能减排实现年度控制目标。较好地完成了市十五届人大二次会议确定的各项目标任务。

    第一，多措并举稳定经济增长

    牢牢抓住实体经济这个稳增长基础。坚持问题导向，分类施策，精准施策，主动服务，认真落实中央、省一系列稳增长政策，及时研究出台支持小微企业发展、促进房地产市场健康发展等政策措施，组织开展“暖企”专项行动。全年争取上级转移支付资金33.6亿元，减免企业税收5.7亿元，拨付财政扶持发展专项资金10.7亿元；充实政策性担保机构国有资本金2.24亿元，担保放大倍率居全省首位；设立企业还贷周转金5.5亿元，协调过桥等续贷资金45亿元；完善财政资金存贷考核办法，协调金融机构动态认领中小企业226户，点对点新增贷款21.4亿元，引导金融机构增加小微企业贷款14.8亿元；设立小微企业创业互助基金，帮助企业实现贷款5000余万元；推动企业直接融资71.5亿元，是上年的3.17倍，1家企业在“新三板”挂牌，27家企业在“新四板”挂牌。坚持尊重历史、面对现实、着眼未来，积极化解了一批地产项目和信贷遗留问题。有效地提振了市场信心。工业经济发展指标企稳回升，净增规上工业企业33户，经济效益综合指数居全省第一。

    牢牢抓住项目投资这个稳增长关键。组织开展了“重大项目提质提效年”活动，完善项目谋划和推进机制。毅远光电LED等117个投资过亿元项目开工建设，精隆电工特种漆包线等92个亿元以上项目建成或基本建成，金昌冶炼厂异地改造等一批重大项目前期工作进展顺利。突出精准招商、板块招商，着力提高招商实效，引进五株、安博、微影光电等一批PCB组团项目，总投资40亿元；新设立外资企业5家；全市在建省外投资亿元以上项目298个，实际到位资金400亿元。持续开展土地房屋征收拆违专项行动，拆除违法建设14.2万平方米，征收房屋70.4万平方米、土地1.5万亩，交付净地1.2万亩，有力保障了项目建设。

    第二，大力促进经济转型升级

    加快调整优化产业结构。深入推进“两化融合”，精心实施“技改三年行动计划”，升级改造传统产业，工业技改投资完成220亿元，增长30%以上，铜产业首位度不断提高。重点发展六大战略性新兴产业，新增企业12家，建成中冠电缆一期等一批重大项目，产值增长20%，电子材料产业规模日益壮大，高端装备制造业渐成气候。现代服务业快速成长。红星美凯龙、雨润中央商场、大润发等一批商业综合体顺利开业；财富广场、铜陵商城、黑天鹅装饰大厦等楼宇经济初具规模；铜都金融商务中心、服务外包基地等服务业集聚区加快建设；渤海商品交易所入驻铜陵，淘宝网“特色中国铜陵馆”建成开馆，新型业态蓬勃发展；天井小镇文化旅游区被评为全省十大文化产业示范园区。

    服务业增加值占GDP比重提高1.4个百分点。现代农业提质增效。建成高标准农田2.53万亩，改造提升标准化蔬菜基地和养殖水面2000亩，耕地流转率居全省第一；新增规模以上农产品加工企业8家、家庭农场67个；油用牡丹、生姜等特色产业发展取得新的突破；夏粮总产量实现“十连增”。

    加快培育发展民营经济。深入实施民营经济促进计划、中小企业希望工程。新增民营企业1750家、个体工商户5080户，每万人拥有民营企业数和个体工商户数居全国前列；民营经济增加值占GDP比重提高1.8个百分点。

    加快建设转型发展平台。政策平台发挥效应。资源型城市转型绩效考评名列全国榜首，节能减排示范市、循环经济示范创建市年度建设任务全面完成；成功争创国家工业绿色转型发展试点市。园区平台完善功能。市经济技术开发区转型发展步伐加快；示范园区具备了大规模承接产业转移落地条件；“圈区管理”金属再生产业园一期建成并通过国家验收，第一口岸转关转检正在国家部委会签；铜官山区现代服务业三个组团初具规模；狮子山开发区跻身省级高新区行列；金桥、大桥开发区扩区获得批准；铜陵蚌埠现代产业园发展框架全面拉开。港口开发有力启动。港口建设完成投资2.5亿元，增长108%。铜陵东港公用航道顺利开通，综合锚地全面建成，港口信息化一期工程、长江干线船型标准化建设如期完成。出台了加快港口经济发展的系列措施，港口货物吞吐量突破7000万吨，增长19.3%。

    加快科技创新驱动转型。大力建设创新载体，成为全省首批创新型试点城市；全社会研发经费占GDP比重2.85%，名列全省前茅；新增国家高新技术企业19家。产学研合作深化，企业创新能力不断增强。新增工信领域国家行业标准9个；新增发明专利授权量145件，每万人发明专利拥有量居全省第三；去年获得省科技进步奖等级、数量均创历史最好成绩。国家智慧城市试点市建设正式启动。创建全国质量强市示范市工作通过省级验收。国家可持续发展实验区、全国节水型社会试点市顺利通过国家验收。

    第三，提质提效推进城乡建设

    交通设施建设提速推进。京福高铁铜陵段全线铺通，铜陵北站及站前路、站前广场主体完工。宁安城际铁路铜陵段基本建成。庐铜铁路启动建设。铜南宣高速进入路面施工阶段。沿新大道顺利完工，省道320、321和坝白路一级路改造全线开工，滨江大道北段、朱永路建设加快推进。铜陵长江三桥开展前期工作。

    城市功能建设提档升级。市体育中心、铜博物馆、城市展示馆和循环经济展示馆等一批重大项目竣工投入使用；新文化馆开工建设。全长3.2公里的滨江生态公园建成开放，昔日脏乱差的滨江岸线成为美丽的铜陵“外滩”；西湖现代农业生态示范园路网、驳岸基本完工；全国规模最大的气象主题公园、天井湖西湖整治等一批生态项目竣工。滨江大道、景观大道、太平湖路、白云路、金岭道等一批城市主干道建成通车；打通了五松道等一批“丁”字路、断头路和微循环道路，北京路下穿涉铁工程顺利完工。亮化106幢楼宇，增添了城市魅力。新（改）建污水管网55公里、燃气管道71.5公里，老城区基本实现雨污分流。临津公园建成开放，大通澜溪老街修缮工程一期完工，东部、南部城区建设稳步推进。

    美好乡村建设亮点纷呈。坚持点线面结合，充分发挥基层干部群众的创造力，高效完成16个省级中心村、20个市级中心村和7条综合示范带建设任务。铜陵县入列全国首批美丽乡村建设标准化试点，凤凰山牡丹花卉入选首批“中国美丽田园”景观，美好乡村已成为我市新的魅力板块。

    第四，强力整治城乡生态环境

    集中开展“三线三边三小”环境综合整治。以治脏、治乱、治污、植绿为重点，组织千人会战，多路出击、多点攻坚，实施重点整治项目336个。铜芜路、铜贵路、顺凤路、翠湖六路、滨江大道、铜都大道、沿新大道等全线焕然一新，蛤蟆岭、罗凤庵、长江大桥等重点区域呈现新貌；依法关停、拆除滨江小码头和非码头设施54个，关闭清场石料矿山21家、取缔小选矿厂（点）136家，提前一年实现煤矿整体退出；率先在全省创建绿色矿山，完成了13个矿山环境整治项目。大力推进森林增长和绿化提升工程，造林绿化2.9万亩，建成森林长廊25.7公里、城市绿道39.9公里，完成或超额完成全年目标任务。

    切实加强重点领域污染治理。全面落实大气污染防治行动计划，实施了七大治理工程，完成工业污染治理、燃煤锅炉淘汰、餐饮油烟整治等治理项目3507个，秸秆焚烧得到有效控制。饮用水水源地保护得到进一步强化，顺安河水质由劣五类提升到三类标准。完成4个重金属污染治理工程。危险废物规范化管理水平居全省前列。建筑垃圾填埋场建成使用，城市生活垃圾无害化处理率100%，保持全省领先水平。

    第五，全面落实各项改革任务

    行政体制改革深入推进。组织编制了权力清单、责任清单和负面清单，发布了市级涉企收费清单，全面清理规范行政审批前置条件。制定了新一轮机构改革方案，完成工商、质监和食品药品管理体制改革。

    经济体制改革继续深化。完善预算编制工作，首次完整编制全口径预算和债务收支计划。整合财政专项资金，改单向资助为竞争性选择。政府购买社会服务项目数和资金额均增长50%以上。“营改增”试点扩大范围。工商注册资本实行认缴登记制，成为全省“先照后证”改革试点市，各类市场主体增长35%。城市基础设施建设PPP试点开始启动。区域通关一体化改革稳步推进，通关成本大幅降低。

    城乡一体化综合配套改革不断深入。基本完成城乡一元化的户籍制度改革。农村土地承包经营权确权登记颁证改革试点进展顺利，农村产权抵押融资保证保险试点进入实质操作阶段，市农村综合产权交易中心挂牌。成为省住房保障金融服务试点市。棚户区改造“房票”安置方式向全国推广。

    社会事业改革取得突破。扩大优质公共资源覆盖面，组建医疗联合体5家、联合办学体4家。“先住院、后付费”诊疗服务模式、校长职级制改革等在全省产生广泛影响。市博物馆、图书馆、文化馆等公益性事业单位理事会完成组建。普济圩农场社会事务完成划转。率先在全省开展警务机制改革，推进机构整合、警力下沉。社区“三减一加强”成效明显，社区事务、考核、挂牌精减50%以上，铜官山区成为全国和谐社区建设示范城区。我市成为首批全国社会组织建设创新示范区。

    第六，持续加强民生和社会建设

    民计民生不断改善。精心实施38项民生工程，投入资金14亿元，全年财政民生类支出85亿元，占财政总支出的80%。惠及面和受益水平均居全省前列，民生工程连续八年处于全省领先位次。坚持以创业带动就业，成功举办首届创业项目大赛，市大学生创业园成为全国创业孵化示范基地，省级创业型城市顺利通过验收。社会保险参保率稳步提高，全面推进城乡大病医疗保险制度，在全省率先开展异地就医双向结算。出台实施了新一轮棚户区改造意见，有效盘活安置房源，春节前3000户以上棚户区居民将迁入新居；集中安置拆迁户1.03万户；改造农村危房1899户；批准发放公积金贷款和商业贴息贷款7.8亿元，改善了3100户中低收入职工家庭住房条件；新开工保障性住房6000套、基本建成4000套。解决渔民生活困难，430户渔民告别以船为家历史。普济圩区域2万职工群众、铜陵县2.7万农村居民饮用水安全问题得到解决。“七老”人员补贴发放到位。城乡低保、农村五保供养标准提高10%以上。

    文明城市创建活动深入开展。广泛开展社会主义核心价值观主题宣传教育活动，志愿服务、铜陵好人评选进入常态化，建成铜陵好人馆，万人拥有中国好人数居全国地级市榜首。组织开展市容环境整治系列行动。大力推进诚信社会建设，建立了诚信“红黑榜”。全国第四届文明城市创建将作为全省首发城市参加“国检”。

    社会事业全面发展。均衡教育深入推进，在全省率先实现全部县区通过义务教育均衡发展国家级验收。铜陵技师学院获得批准。公共文体活动蓬勃开展，建成农民文化乐园10个，大通镇入列第六批中国历史文化名镇，我市成为全国全民健身示范市。市立医院晋升为国家三级综合性医院。单独两孩政策得到落实。市社会福利院基本建成，市养老康复中心主体工程完工，全市养老床位增长15%。残疾人工作示范县、区在全省率先实现全覆盖。顺利完成第九届村委会换届选举。积极开展双拥工作，圆满完成国防动员指挥演练。安全生产形势持续稳定。扎实开展守护平安系列专项行动，认真排查化解信访积案和突出问题，成为全省社会管理综合治理先进市。深入推进“六五”普法，成为全国“六五”普法中期先进市。社会科学、新闻出版、广播影视、文学艺术、统计、对台、外宣、档案、地方志、供销、地震、消防、兵役等工作得到加强，民族宗教、外事侨务、妇女儿童、红十字等事业取得新成绩。

    与此同时，我们扎实开展群众路线教育实践活动，切实加强政府自身建设。认真践行“三严三实”，严格落实“三张清单”工作法，解决了一批群众反映的实际问题。行政审批项目数全省最少，政务服务标准化工作领先全省。“12345”市长热线、行风热线、市民论坛累计受理群众诉求和建议2.4万件，做到事事有落实、件件有答复。自觉接受人大法律监督、工作监督和政协民主监督，办结人大代表建议233件、政协提案282件。严格执行中央八项规定和省、市委规定，“三公”经费支出下降30%；严格控制楼堂馆所建设，机关办公用房、党政干部住房清理取得阶段性成效；严肃开展政策性安置房清查工作，清退住房5.78万平方米、资金3500万元。认真开展政府系统管理问题集中查摆活动，强化重点领域、关键环节的行政监察、审计监督，查处了一批违法违纪案件。

    各位代表！过去的一年，我们科学把握发展大势，主动适应经济发展新常态，有效应对各种风险和挑战，实现了经济持续健康发展和社会和谐稳定，成绩确实来之不易。这是省委、省政府和中共铜陵市委正确领导的结果，是全市人民同心同德、奋力拼搏的结果，必须充分肯定、倍加珍惜。在此，我代表市人民政府，向全市广大工人、农民、知识分子、干部、离退休老同志，向驻铜解放军指战员、武警官兵和政法干警，向各民主党派、各人民团体和社会各界人士，向关心、支持铜陵发展的省直各部门和海内外友好人士，向在我市创业的投资者、建设者，表示衷心的感谢！

    回顾过去的一年，我们也清醒地认识到，作为资源型城市，我市发展面临诸多困难和挑战。经济下行压力持续加大，投资增速趋缓，房地产市场调整影响不断扩大，有色、化工、钢铁等行业市场低迷，要素成本上升，企业生产经营困难增多；财政收入增速放缓，刚性支出不断增加，地方债务风险正在积聚；经济结构不优，传统产业占比过高，战略性新兴产业发展快而不大，现代服务业发展滞后，引领升级的大项目、好项目不多，经济转型尚处在负重爬坡阶段；工程建设管理还存在不少薄弱环节，城市功能不强，还欠账、补短板、提品质任务艰巨；资源环境约束加剧，生态文明建设亟待加强；推进社会治理创新面临新挑战，维护社会稳定压力较大。政府基础管理不够到位，工作谋划和落实需要改进，依法行政和服务群众能力有待提高。少数公务人员作风不正、为官不为、为政不廉，加强作风建设、提升效能任重道远。对此，我们一定高度重视，切实加以解决。

    二、2015年重点工作安排

    今年是全面深化改革的关键之年，是全面推进依法治市的开局之年，也是全面完成“十二五”规划的收官之年，做好今年工作至关重要。当前，宏观经济形势仍然错综复杂，我国经济发展进入新常态，增长速度变化，结构调整优化，发展方式转向，发展动力转换，发展的条件和环境发生着深刻而复杂的变化。新老问题叠加，内外矛盾交织，给我们带来严峻挑战。同时更应当看到，我们仍处于大有可为的重要战略机遇期没有改变，经济发展总体向好的基本面没有改变。国家建设长江经济带，为我市加快转型发展带来了历史性机遇；宏观政策导向与我市发展需求高度契合，为我们提供了新的政策利好；内外交通格局大变化，将有力拓展新的发展空间；全面深化改革，市场活力和内生动力将进一步释放。我们要认识新常态，适应新常态，引领新常态，牢固树立转型发展、清洁发展、安全发展、和谐发展意识，牢固树立转方式、调结构、促升级意识，牢固树立开放合作意识，牢固树立“绿水青山就是金山银山”意识，牢固树立尊重规律、注重统筹、主动作为意识。我们要顺势而为，乘势而上，对应上级政策发力，对应群众需求发力，对准县区园区发力，对准港口岸线发力，对准城乡一体发力，以我们的辛苦指数，换取群众的幸福指数，提升铜陵的发展指数。

    做好今年的政府工作，我们必须全面贯彻党的十八大、十八届三中、四中全会，习近平总书记系列重要讲话和中央、省委省政府系列会议精神，认真落实市委决策部署，坚持稳中求进工作总基调，以全面深化改革、全面推进法治建设为引领，以提高经济发展质量和效益为中心，着力调结构、促转型，着力惠民生、促和谐，着力优作风、促落实，加快城乡一体、产城一体、港城一体化进程，确保全面完成“十二五”目标，为干好“十三五”打下坚实基础。

    今年经济社会发展的主要预期目标是：全市生产总值增长8.5%左右；财政收入增长5%左右；全社会固定资产投资增长10%以上；社会消费品零售总额增长12%以上；城镇、农村居民人均可支配收入分别增长8.5%和9.5%以上；城镇登记失业率控制在3.8%以内；居民消费价格涨幅控制在3%左右；确保完成省政府和国家节能减排示范市确定的年度目标任务。

    重点抓好五个方面工作：

    （一）稳企业、抓投入，全力促进经济稳定增长

    深入实施“暖企行动”。稳定并完善各项帮扶措施，发挥政策叠加效应。继续开展项目前置审批条件、市场准入限制等清理工作。落实涉企收费清单，做到清单之外无收费；落实定向降税、普遍性降费等政策，切实减轻企业负担。加强政银企合作，进一步壮大政策性国有担保机构资本金规模，用好应急周转金、过桥资金，扩大小微企业创业互助基金受益面。各商业金融机构认领支持企业数、贷款额要高于去年，切实化解中小企业资金瓶颈制约。完善财政资金专项扶持政策，抱团取暖，切实帮助企业解决实际困难。

    积极支持企业做大做强。深入实施百亿企业服务工程、中小企业希望工程和民营经济促进计划。支持铜陵有色整体上市，向世界500强迈进；推动铜化集团做精做优；促进中小微企业发展壮大。鼓励上市公司利用“壳”资源，引进战略投资者，进一步优化重组、加快发展。全年净增规上工业企业27家，新发展民营企业1500户、个体工商户5000户；规上工业增加值增长10%，民营经济占经济总量比重提升1.2个百分点。鼓励企业走出去、引进来，加大外贸企业孵化支持力度，外贸进出口增长5%。

    强力推进各类项目建设。发展是硬道理，项目是硬发展。继续坚持经济工作项目化、项目工作责任化，实施“重大项目谋划建设年”活动，健全项目指导、谋划、调度、推进和实现机制，破解用地、资金、环境等要素制约，确保项目建设顺利推进。全年安排亿元以上项目427个，其中新开工80个、竣工65个。完成工业投资400亿元，城乡建设投资200亿元以上，现代服务业重点项目投资74亿元。促进金昌冶炼厂异地改造、皖能电厂六期等一批重大项目开工建设，推动新兴际华低氧铜杆、五株电路等一批重大项目加快建设。瞄准国家重点支持的方向，在工业发展、重大基础设施、节能环保、现代物流等方面谋实一批全局性、战略性的重大项目，加速谋划项目的转化。继续开展土地房屋征收拆违专项行动，为项目建设提供用地保障。

    切实加大招商引资力度。紧盯长三角、珠三角等重点区域和央企、名企，实行精准招商。密切关注产业加速转移新动态，围绕铜基新材料、节能环保、先进装备制造、医药和精细化工、现代服务业等，实行“点对点”式承接，注重产业链招商、板块招商、以商引商，提高招商引资精准度。进一步强化县区、园区招商主体责任，完善激励措施，健全服务机制，促进项目落地，各县区、园区引进超10亿元重大项目2个以上。力争三峡氨基酸、新能源汽车等10亿元以上重大项目签约落地，全年实际利用省外资金与外商直接投资均增长10%以上，引进5亿元以上项目34个以上。

    着力优化经济发展环境。牢固树立“实体兴，铜陵兴；工业兴，铜陵兴”理念。完善金融服务体系，支持银行、证券、保险等金融机构健康发展，鼓励金融产品创新；加强与徽商银行总行、省建设银行等金融机构的战略合作，全年新增贷款75亿元以上。加大直接融资力度，支持发展产业基金和创业投资基金，新增“新三板”挂牌企业5家、“新四板”挂牌企业10家，推进市建投公司等企业债申报发行工作，全年直接融资50亿元以上。积极争取上级支持，全年对上争取政策资金35亿元。规范土地管理，盘活用地存量，提高投资强度，保障发展需求，集约节约用地，全年盘活闲置土地2000亩以上。

    注重防范经济运行风险。理顺政府债务管理体制，将政府债务纳入全口径预算管理，合理安排融资规模和结构，完善借用还机制，确保政府债务总体可控。防范化解企业债务风险，一方面，强化金融机构共兴共荣意识，保障企业资金链安全；另一方面，强化社会信用契约意识，共同改善信用环境。严格规范民间融资，严厉打击非法集资，维护金融秩序稳定，避免出现区域性、系统性金融风险。

    （二）调结构、促转型，加快产业升级步伐

    构筑多元发展多极支撑的产业体系。以技术改造、信息化、智能化运用为抓手，大力促进传统优势产业向精深加工延伸、向终端产品拓展、向质量品牌提升。推进“技改三年行动计划”，实行百项技改对标“销号”，技改投资增长15%。发展壮大战略性新兴产业，增强铜基新材料产业核心竞争力，推动先进装备制造、节能环保、新能源和文化创意等产业做大做强，促进企业组团发展、产业集群发展，战略性新兴产业产值增长15%以上。大力发展研发设计、科技服务、节能环保服务等生产性服务业；加快服务业集聚区及大型商业综合体建设，扩大商贸服务业辐射功能；培育电子商务、服务外包等新型业态，推动健康服务业医养一体化，引导社会力量参与养老服务业，促进文化、旅游、体育等产业融合发展。服务业增加值增长8%。积极培育信息消费、旅游休闲等新兴消费热点，促进房地产市场平稳健康发展。

    促进县区园区发展提效升级。把发展县域经济放在更加突出的位置，完善县域经济发展措施，引导县区因地制宜发展产业，做大做强，立足全省，争先进位。推动开发园区培育首位产业，壮大特色产业，发展产业集群，当好转型发展排头兵。支持铜陵县进入全省县域经济发展先进行列，鼓励铜官山区争创国家服务业综合改革试点区，引导郊区加快大通历史文化旅游资源开发，推进狮子山区高新区提档升级。市经济技术开发区要加快铜基新材料、PCB等产业园建设，促进产城融合。示范园区要加快重大项目落地见效，确保港口物流、再生资源等百亿元产业集群建设有实质性进展。

    强化转型平台支撑功能。大力推进国家节能减排示范市、循环经济示范创建市建设，实施五大类节能减排示范项目、102个“六化”项目，争创国家循环经济教育示范基地。统筹抓好资源型城市转型试点市、国家工业绿色转型发展试点市建设。发挥“圈区管理”金属再生产业园综合效应，争取进口废七类集装箱转关转检第一口岸获批。积极创造条件，申报综合保税区、铜期货交割仓库。建设PCB国家级质检中心。推进智慧城市建设，建成全市统一的云计算数据中心。

    加速港城一体化建设进程。抢抓长江经济带建设机遇，编制完善铜陵港总体规划和重点港区控制性详规。强力整治沿江岸线环境，完成从大通到笠帽山全线整治任务，拆除小码头和非码头设施，同步推进生态建设，进一步彰显工贸港口城市风貌，为临港经济大发展留足空间。加快横港、长山、永丰等港区10个万吨级码头建设，推进有色金属物流园、顺通物流园、国定快递分拣园等一批项目建设，探索地主港模式，促进临港经济跨越式增长，尽快实现亿吨大港目标，打造长江中下游重要的水运转港节点城市。积极谋划推进跨江发展，推动建立合肥铜陵现代工业走廊；积极谋划建设公、铁、水衔接顺畅的立体交通网络，着力构建区域综合交通枢纽；积极谋划推进长江铜陵段综合治理、顺安河等内河航道改造和铜陵三桥、过江隧道、江北港储中心等重大项目，努力形成皖江开发开放新高地，提升城市能级。

    加快创新驱动发展。推进省创新型试点城市建设，强化企业创新主体地位，新增国家高新技术企业15家、省级工程技术研究中心3个，全社会研发经费投入占地区生产总值的比重保持全省领先。办好中科院皖江新兴产业技术发展中心等产学研平台，探索技术转让协同创新、科技金融结合创新模式。积极鼓励大众创业、万众创新，创新人才引进和培养机制，扩大科技创新股权激励试点，力争引进一批科技创新团队和行业领军人才。加强知识产权创造、运用与保护，新增发明专利申请量1500件，发明专利授权量150件。坚持质量强市，加快创建国家质量强市示范市。

    （三）强功能、提品质，统筹推进城乡一体建设

    着力促进内外交通大顺畅。今年，全市人民期盼的京福高铁、宁安城际铁路将相继投入运营，铜陵将迎来高铁时代。我们要主动对接服务，确保铜陵北站及站前广场年中投入使用，京福高铁公路连接线年内建成。积极配合做好庐铜铁路建设。主动服务铜南宣高速建设，力争年内建成通车。贯通省道320、321、坝白路、朱永路，完成滨江大道“黑色化”改造。

    着力增强城市综合承载能力。围绕拉开框架、完善功能、提升品位、塑造特色、加强管理，加快建设宜居宜业的山水园林城市。完善新一轮城市总体规划、土地利用总体规划。完善市政设施。新（改）建天山大道、谢垄路等主次干道，完成北京路下穿工程，继续打通一批“丁字路”、断头路和微循环道路，新建一批停车场，不断缓解主城区交通拥堵状况。开工建设主城区污水管网截污工程，新（改）建供水管网35公里。建成新图书馆、新文化馆、市老年活动中心，改造五松山剧场及一批体育健身设施，完善公共服务功能。改造提升30个以上老旧小区，整治城市主次干道风貌。统筹发展城乡公交，做好城市公交与镇村公交的对接，推进公交智能化建设。加快新区建设，开工市一中西湖校区、市儿童医院和中职实践基地等项目，建成西湖现代农业生态示范园，配置优质公共资源，聚集人气，带动区域开发建设。加快东部城区配套建设，提高综合承载功能；强化南部城区基础设施和生态建设，拉开城区框架。

    着力抓好城乡生态环境整治。实施倒逼机制，淘汰落后产能，减少污染物排放。推进大气污染防治“六大行动”，压能耗、降扬尘、控车辆、迁企业、抓减排、增绿化，重点整治南部城区、码头、矿区扬尘污染，完善治超载、治泼洒长效机制，巩固石料矿山、小选矿厂整治与秸秆禁烧成果，PM10平均浓度力争下降5%。深入推进“三线三边三小”整治，基本实现铁路、公路、江河沿线的整齐清洁常态化。完成城区黑砂河、幸福渠、七坝沟“三大水系”治理任务。扎实推进森林增长和绿化提升工程，新增造林绿化面积1.93万亩，新增和改造提升绿化面积200万平方米，建成森林长廊示范段5公里、城市绿道30公里，恢复矿山植被3000亩。积极争创国家森林城市。加强环境执法，保持对污染的严管态势，铁腕治污、铁面执法、重拳出击，加大曝光和处罚力度，既要金山银山，也要绿水青山和蓝天白云。

    着力促进美好乡村扩面提质。围绕村庄美，把握好“记得住乡愁”的规划环节，发挥好农民主体作用，结点连线成片，建好16个省级中心村、20个市级中心村。健全美好乡村长效管理机制，巩固建设成果，放大示范效益。围绕生活美，大力发展都市现代农业。积极推进农业产业化经营，新增家庭农场30家；加大特色农产品开发力度，发展牡丹深加工和白姜产业；促进专业蔬菜基地和水产养殖基地建设。围绕乡风美，深入推进农民文化乐园建设，打造优秀乡土文化和现代文明有机融合的和谐乡村。

    （四）抓重点、求突破，全面深化各项改革

    深入推进城乡一体化综合配套改革。完善和落实城乡一元化户籍制度，有序推进常住人口市民化。做好住房保障金融服务试点工作。积极稳妥推进农村产权制度改革，全面完成农村土地承包经营权确权颁证，加快推进农村房屋确权颁证，开展农村小型水利工程确权颁证试点，实现农村集体建设用地、宅基地、林地确权颁证常态化。推进农村集体“三资”股权量化改革。促进农村产权交易规范运转。

    积极推进经济体制改革。深化财税体制改革，认真落实新《预算法》，加快建立全面规范、公开透明的预算制度。进一步扩大“营改增”范围，全面完成“营改增”任务。深化投融资体制改革，积极稳妥地开展PPP试点，引导社会资本进入公共服务和市政基础设施等重点领域。深化国有资产管理体制改革，提高国有资本收益上缴比例。加强企业负责人薪酬考核，规范履职待遇和业务支出。坚持彻底改、改彻底，积极稳妥推进国有企业改革，完善法人治理结构。推进天然气等资源性产品价格改革，完善市场决定价格机制。

    稳步推进行政体制改革。完成新一轮政府机构改革，推进政府职能转变。制定市、县（区）、乡镇三级政府事权划分标准，明确权力边界。全面落实“三项清单”制度，确保权责法定。探索相对集中行政许可权，制定事中事后监管办法，实现由“重批轻管”向“宽进严管”转变。深化工商注册登记“先照后证”试点，激发社会创业活力。启动新一轮公共资源交易及监管体制改革。完成党政机关及参公事业单位公务用车改革。

    扎实推进社会事业改革。深化医疗卫生体制改革，加强医疗、医保、医药改革联动，建立基本药物采购、配送、销售和集中支付运行机制，市二级以上公立医院药品实行集中配送。推行“先住院、后付费”诊疗服务模式，扩大医疗联合体组建面。深化教育综合改革，扩大校长职级制改革试点，推行教师注册制度，探索管、办、评分离，推动优质教育资源向县区、新区拓展。积极推进事业单位改革，扩大事业单位法人治理结构试点。稳步实施机关事业单位养老保险制度改革。巩固社区“三减一加强”成果，做好城市社区居委会换届选举，推动形成社区居民广泛参与的多元共治机制。

    （五）促均等、惠民生，提升居民幸福指数

    扎实推进民生工程。坚持守住底线、突出重点、完善制度、引导舆论的基本思路，统筹做好教育、就业、医疗、养老、住房等群众关心的民生事项。今年，继续组织实施38项民生工程，加快推进城乡基本公共服务均等化，让人民群众更多更好地分享改革发展成果。

    大力推进棚户区改造。开展“棚户区改造推进年”活动。本着积极而为、量力而行、尊重民意、循序渐进的原则，将棚户区改造与保障房、安置房建设相结合，与消化存量、激活需求相结合，推进横港扫把沟、露采、东村、狮子山老城区等成片棚户区改造，支持有色公司、铜化集团等企业优先改造，完成改造4200户，实现棚户区地区大变样。加大安置房的建设和搬迁力度，再安置1万户以上。

    不断完善就业服务和社会保障体系。争创国家创业型城市，完善创业就业政策体系，加大创业孵化、技能培训和就业援助等工作力度，扎实做好高校毕业生、农村转移劳动力等重点群体就业工作，全年新增城镇就业岗位2万个。建立健全保障农民工工资支付的长效机制，维护劳动者合法权益，构建和谐劳动关系。扩大社会保险参保覆盖面，增强基本医疗保险功能，增加大病保险报销范围，提高被征地农民和城乡居民养老待遇水平。积极争创全国“救急难”工作试点市，提高城乡低保、五保供养、医疗救助、临时救助水平，把困难群众的基本生活底线兜住兜好。

    统筹发展各项社会事业。加强教育信息化建设，加大“名教师”培养力度，提高教育现代化水平。实施学前教育三年提升计划，推进义务教育阶段学校标准化建设，迁建市特殊教育学校。深化产教融合、校企合作，推进高职中职衔接，组建职业教育集团，建好技师学院，提高技能型人才培养质量。加快发展高等教育，支持铜陵学院建设地方应用型高水平大学。增强医疗服务能力，支持市立医院创建三甲医院，建成市人民医院外科楼，建设一批特色专科，打造皖中南医疗服务中心。做好人口计生工作，争创全国幸福家庭示范市。推动文化品牌建设，编制大工山—凤凰山古铜矿遗址保护等规划，完成大通古镇修缮保护工程，弘扬青铜文化和地方特色文化。推进市级体育场馆市场化运营，广泛开展全民健身运动。加快发展妇女儿童和老龄事业。落实残疾人权益保障制度。大力发展福利慈善事业。

    持续提升文明创建水平。深入开展社会主义核心价值观教育，推进铜陵好人评选、道德讲堂、志愿服务等活动经常化，加强诚信制度化建设，健全市政设施管养体制机制，推动市容环境城乡联创、文明成果城乡共享，力争跻身全国第四届文明城市行列。

    切实加强社会治理创新。增强社会法治观念，推进法治社会建设。实施社区服务提升工程，建成社会服务管理信息化平台，打造一批城市精品社区、农村新型社区。创新社会组织管理，实现工商经济类协会和商会与政府机关脱钩，积极培育公益类、社区类等社会组织，支持其平等参与社会治理。深入推进平安铜陵建设，完善打防控一体的社会治安防控体系，依法打击各类违法犯罪行为，增强人民群众安全感。

    强化信访问题源头治理，妥善化解信访热点和遗留问题。严格落实新《安全生产法》，防范和遏制重特大安全事故发生。强化食品药品安全监管。提高保障公共安全和应对突发事件能力。

    各位代表！完成“十二五”规划是今年的一项重要任务。我们要加油鼓实劲，对标抓落实，圆满实现预期目标。同时，要立足新常态，顺应新期待，抢抓新机遇，深谋发展战略，细研建设布局，坚持“多规合一”，高起点编制“十三五”规划，精心绘就转型发展的新蓝图。

    三、加强法治政府建设

    面对新的形势，新的任务，我们必须按照党的十八届四中全会部署，忠实履行宪法和法律赋予的神圣职责，加快建设职能科学、权责法定、执法严明、公开公正、高效廉洁、守法诚信的法治政府。

    （一）依法履行政府职能。政府机关必须坚持“法定职责必须为、法无授权不可为”，勇于负责、敢于担当，坚决纠正不作为、乱作为，坚决克服懒政、怠政，坚决惩处失职、渎职。各级政府及其部门不得法外设定权力，不得减少本部门法定职责。正确处理好政府与市场、与社会的关系，强化政府基本公共服务均等化职责。大规模、多形式开展干部的法律培训，增强法治意识，提高法治素养，自觉运用法治思维和方式推动改革、促进发展、维护稳定。

    （二）提升依法决策水平。规范重大决策行为，把群众参与、专家论证、风险评估、合法性审查、集体决策作为必经程序，以程序的缜密，保证决策的科学。严格决策审查流程，进一步完善政府法律顾问制度，建立行政机关内部重大决策合法性审查机制，做到重大决策不经合法性审查不决定，重大政策不经合法性审查不出台，重要协议不经合法性审查不签署。强化决策责任落实，健全重大决策终身责任追究制度和责任倒查机制，做到失责必追究。增强决策的执行力，推动部署的任务掷地有声，及时问责不落实的人和事。完善决策后评价机制，健全政府规范性文件立、改、废机制，做好地方立法权相关准备工作。

    （三）增强行政执法能力。依法界定市、县（区）两级执法分工和责任，推进执法重心下移。进一步理顺城管执法体制，在市场监管等领域推进综合执法，提高执法效率。各级政府都要规范执法行为，严格执行执法人员持证上岗和资格管理制度，严格执法程序，严肃落实行政执法责任，细化、量化行政自由裁量权，杜绝随意执法、人情执法，做到严格、规范、公正、文明执法。

    （四）严格约束行政权力。自觉接受人大及其常委会的法律监督、工作监督，执行人大决议，重大决策出台前及时向人大报告。主动接受政协民主监督，加强协商，有事多商量，遇事多商量，做事多商量。认真办理人大代表建议和政协提案，不断提高办理质量。主动听取民主党派、工商联、无党派人士的意见，充分发挥工会、共青团、妇联等人民团体的作用，认真听取离退休老同志的意见和建议，高度重视社会公众和舆论的监督。加强行政监察、审计监督和政务督查，改进政府内部监督，完善纠错问责机制。坚持公开为常态、不公开为例外，不断扩大政务公开范围、细化公开内容，做到决策公开、执行公开、管理公开、服务公开、结果公开，让权力在阳光下运行。

    （五）着力强化政府管理。开展“政府管理提升年”活动。按照“理顺管理体制、强化基础管理、突出重点管理、引导示范管理”的思路，完善文书、档案、会议、活动等基础管理规程和标准，制定人财物和政府投资项目全过程管理规范，总结推广典型经验，发挥示范引领作用，改变管理粗放、程序混乱、责任不清、成本失控等现象，促进政府管理科学化、标准化和规范化。

    （六）坚持依法从严治政。严格执行中央八项规定和省、市委有关规定，牢固树立过“紧日子”的思想，开展机关事业单位“吃空饷”问题集中治理工作，确保财政供养人员只减不增，确保“三公”经费只减不增。把权力关进制度的笼子，用制度管权管人管事。认真履行党风廉政建设主体责任，以锲而不舍、驰而不息的决心，不断提高拒腐防变和抵御风险能力。始终保持反腐败的高压态势，以“零容忍”的态度坚决查处各类违纪违法行为。

    各位代表！做好今年工作，我们要以昂扬的精神状态，强烈的使命担当，适应新常态，开拓新局面。我们要深入持久地加强作风建设，认真开展“三严三实”专题教育，巩固拓展教育实践活动成果，深、实、细、严、快地推进工作，形成领着干、推着干、比着干的干事氛围。我们要强化责任担当，在全市上下形成主要领导负总责、分管领导抓分管、主管部门抓行业、县区园区抓区域的责任体系，以责任的落实推动工作的落实，以执行的到位推动发展的跨越。我们要始终保持为民服务的公仆情怀，与人民心心相印、与人民同甘共苦、与人民团结奋斗！

    各位代表！

    迎接新挑战任务艰巨，开启新征程使命光荣。我们一定不负人民重托，精于谋划，善于创业，真抓实干。让我们在中共铜陵市委的坚强领导下，团结拼搏，奋发有为，谱写全面深化改革、全面依法治市、加快转型发展的新篇章，为建设幸福美丽新铜陵而努力奋斗！
